# Association of FOBT-assessed faecal Hb content with colonic lesions detected in the Florence screening programme

**DOI:** 10.1038/sj.bjc.6603534

**Published:** 2007-01-09

**Authors:** S Ciatto, F Martinelli, G Castiglione, P Mantellini, T Rubeca, G Grazzini, A G Bonanomi, M Confortini, M Zappa

**Affiliations:** 1 Centro per lo Studio e la Prevenzione Oncologica, Viale Volta 171, 50131 Firenze, Italy

**Keywords:** colorectal cancer, faecal occult blood testing, FOBT, screening

## Abstract

We assessed the correlation between quantitative results of immunological faecal occult blood testing (I-FOBT) and colonic lesions (191 colorectal cancers, 890 adenomas) detected at colonoscopy in 2597 FOBT+ (cutoff 100 ng ml^−1^ Hb) subjects. At univariate analysis, a higher average faecal Hb content was significantly associated with male gender (*P*=0.003), age (*P*=0.02), and colonoscopy findings (*P*=0.000). Among adenomas, higher faecal Hb content was significantly associated with size (*P*=0.0000), presence of severe dysplasia (*P*=0.0001), presence of villous component (*P*=0.0002), and location in the left colon (*P*=0.003). At multivariate analysis adjusting for potential confounders, age (*P*=0.03), size (*P*=0.0000), and location in the left colon (*P*=0.0005) were confirmed as having an independent association with higher faecal Hb content. Immunological FOBT is confirmed to be a specific screening test to detect cancer and adenoma, with a low positivity rate (3.7%) and a high positive predictive value (41.5%). Faecal Hb content is significantly higher for those lesions (cancer and high-risk adenomas) screening is aimed at detecting.

Screening by faecal occult blood testing (FOBT) has been shown to be effective in reducing colorectal cancer (CRC) mortality (Mandel *et al*, 1993; Selby *et al*, 1993; Saito *et al*, 1995; Hardcastle *et al*, 1996; Kronborg *et al*, 1996; Castiglione *et al*, 1997; Bertario *et al*, 1999; Mandel *et al*, 2000), and population-based screening is currently recommended by the European Community (Advisory Committee on Cancer Prevention, 2000) and is under implementation in several countries, including Italy (Zorzi *et al*, 2006) (www.osservatorionazionalescreening.it).

Screening efficacy was demonstrated for guaiac-based FOBT, but the high diagnostic performance of immunological FOBT assays (I-FOBT) has recently justified their adoption in current screening practice (Saito *et al*, 1995; Bertario *et al*, 1999). The Florence FOBT population-based screening programme, run by the Centro per lo Studio e la Prevenzione Oncologica (CSPO) since 1982, was confirmed to be effective by means of a case–control study (Zappa *et al*, 1997): adjusted odds ratio for death from CRC for screened *vs* not screened subjects was 0.60 (95% CI: 0.4–0.9). The programme shifted from guaiac to I-FOBT in 1995, after favourable comparison studies with guaiac FOBT (Castiglione *et al*, 1996, 1997, 2000) and positivity cutoff was set at a 100 ng ml^−1^ Hb level (Castiglione *et al*, 2002).

Faecal occult blood testing screening has often been criticised for its limited specificity, Spurious guaiac-based FOBT positivity has not been eliminated with the introduction of I-FOBT, owing to non-neoplastic intestinal bleeding, and the efficacy of FOBT screening has been claimed to be, at least partially, the effect of a large cumulative use of colonoscopy, prompted by spurious FOBT positivity at repeat screening (Lang and Ransohoff, 1994; Ederer *et al*, 1997).

The use of quantitative I-FOBT allows for a precise assessment of faecal Hb content and for assessing the association of Hb content to different types of screen-detected colonic lesions. The aim of the present study was to review a large consecutive series of subjects with positive I-FOBT undergoing diagnostic assessment within the Florence screening programme, in order to assess whether I-FOBT positivity is mostly non-neoplastic and just offers a chance for a screening colonoscopy, or if abnormal Hb content is specifically associated to the presence of CRC and high-risk adenomas.

## MATERIALS AND METHODS

The study analyses a consecutive series of FOBT+ subjects, invited and complying to diagnostic assessment in the Florence screening programme for colorectal cancer. The detailed features of the screening programme, inviting residents in the 50–70 years age group to biennial FOBT, have been described elsewhere (Castiglione *et al*, 1996, 2000; Zappa *et al*, 1997).

A single sampling on a single bowel movement is currently analysed, without any dietary restriction. Immunological faecal occult blood testing used in the study was OC-Hemodia, developed with the OC-Sensor (Eiken Chemical Co, Japan) instrument. The assays is based on the flocculation reaction between human HbA and multiple monoclonal anti-HbA latex-adsorbed antibodies. HbA concentration is measured by reading flocculation as an optical change (increased adsorbance at 660 nm), compared to a standard calibration curve. The inter-series coefficient of variation in our lab was 3.3% (156±5.2) at a low and 3.8% at a high (601±22.9) Hb level.

The current positivity cutoff prompting diagnostic assessment was 100 ng ml^−1^ Hb. From December 1996 to September 2005, screened FOBT+ subjects were 3029 of 81 047 (3.73%). Compliance to diagnostic assessment in the study period was 85.7% (2597 of 3029). Assessment was based on total colonoscopy, or on incomplete colonoscopy plus double contrast X-ray for those subjects in whom total colonoscopy was not possible. Subjects with negative colonoscopy were re-invited to screening after 5 years until 2004 and after 10 years thereafter.

Data available for each case were age and gender, quantitative FOBT Hb content (ng ml^−l^), and colonoscopy outcome (cancer, adenoma, other benign lesion, negative). In subjects with multiple findings (e.g. other benign lesions+adenoma, adenoma+CRC), only the most severe lesion was considered for the study purpose. Adenomas were further categorised by size (<1, 1–2, >2 cm), presence/absence of severe dysplasia, histological features (tubular, villous), and site (right/trasverse or left colon): in presence of multiple adenomas, the larger and/or that with the most severe features were considered for the study purpose.

We assessed average FOBT Hb content (ng ml^−l^) by age and gender, by colonoscopy outcome, and according to special conditions (presence of haemorroids, diverticula) which might be associated to non-neoplastic bleeding and were considered separately from colonoscopy outcome. Average FOBT Hb content was also determined by adenoma subcategories.

Differences between observed results were checked by the *χ*^2^ test, differences between means were checked by the *t*-test and analysis of variance (ANOVA), while Mantel–Haenszel test was used to check counfounding effects. A logarithmic transformation of Hb values was performed, as the Hb histogram was not normally distributed. Statistical significance was set at a *P*<0.05 level. The STATA8 statistical package was used.

## RESULTS

Overall, 2597 FOBT+ subjects (1109 women, 1488 men) were assessed by colonoscopy from December 1996 to September 2005, and were eligible for the study. Age ranged from 48 to 76 years (median age=62). Complete colonoscopy rate in the study period was 82.2% (2137 of 2597).

Colonoscopy detected CRC in 191 subjects (7.3%), adenoma in 890 (34.2%), benign lesion other than adenoma in 985 (37.9%), and was negative in 531 subjects (20.4%). A significantly higher detection rate was observed in men as compared to women for both CRC (8.5 *vs* 5.7%, *χ*^2^_df=1_=7.12, *P*=0.008) and adenoma (39.7 *vs* 26.8%, *χ*^2^_df=1_=47.04, *P*=10^−6^). Increasing age was significantly correlated to CRC (*χ*^2^ for trend_df=1_=6.23, *P*=0.01) and adenoma (*χ*^2^ for trend_df=1_=5.23, *P*=0.02) detection rate. The presence of haemorroids was recorded in 742 subjects (28.5%) and that of diverticula in 598 subjects (23.0%).

Table 1 shows average Hb content observed in studied subjects according to patient characteristics and most severe colonoscopy outcome. Significant differences were observed by gender (*P*=0.003, *t*=−2.90, df=2595) and age (*P*=0.02, *F*=2.75, df=4, 2592), with a peak in the 60–64 years age group. A trend of increasing average Hb content was evident according to colonoscopy outcome, from negative, through benign or adenoma, to CRC findings. Differences were statistically significant (ANOVA, *P*=0.000, *F*=83.42, df=3, 2593). Average FOBT Hb content was higher for cancers located in the left (*N*=135) as compared with right colon (*N*=50, data missing in six cases), but the difference did not reach statistical significance (816.3 *vs* 769.0 ng ml^−1^, *P*=0.98, *t*=0.01, df=183). Out of 890 subjects with adenomas as the unique or most severe lesion, 744 adenomas were detected in the left, and 135 in the right colon (data was missing in 11 cases). Maximum size was <1 cm in 341, 1–2 cm in 279, and >2 cm in 270 cases. Severe dysplasia was reported in 175, and villous component, in 487 cases. A substantial association was evident between adenoma subcategories, severe dysplasia being associated with size (<1 cm=6.7%, 1–2 cm=17.9%, >2 cm=37.7%, *χ*^2^_df=2_=92.64, *P*=10^−6^), and increasing size being associated with villous component (<1 cm=32.5%, 1–2 cm=56.2%, >2 cm=81.1%, *χ*^2^_df=2_=143.80, *P*=10^−6^). Association between severe dysplasia and villous component (tubular=6.9%, villous=30.1%) was apparent, owing to the confounding effect of size (Mantel–Haenszel test, *χ*^2^_df=1_=33.33, *P*=0.000). When adenoma were classified as advanced (larger than 9 mm, or with villous or tubulo-villous histological pattern (>20%), or with high-grade dysplasia) or not advanced, Hb content was significantly higher for advanced adenomas (*N*=669, average 550 ng ml^−1^, median 356 ng ml^−1^) as compared with not advanced ones (*N*=221, average 385 ng ml^−1^, median 222 ng ml^−1^). Larger adenomas were observed in the left as compared with right colon (*χ*^2^_df=2_=6.27, *P*=0.04) (data not shown). Apart from colonoscopy outcome, no positive association with higher Hb content was observed for the presence of haemorroids or diverticula.

Table 2 shows differences in FOBT Hb content according to adenoma subcategories. A significant positive association was evident for increasing size (*P*=0.0000, *F*=31.58, df=2 887), presence of severe dysplasia (*P*=0.0001, *t*=−3.84, df=888), and presence of villous component (*P*=0.0002, *t*=−3.78, df=888). Weaker association was recorded for left *vs* right colon site (*P*=0.0037, *t*=2.90, df=877).

Table 3 shows multivariate analysis of the association of different patient characteristics and colonoscopy outcome to average FOBT Hb content among subjects with adenoma. A significant association was evident only for adenoma size (*P*=0.000) and for localisation in left colon (*P*=0.01), and a borderline association was observed for age (*P*=0.05). After adjusting for interactions, results did not change. The model was thus limited to variables with significant association, and age (*P*=0.03, F=2.63, df=4871), size (*P*=0.0000, *F*=29.29, df=2, 871), and location in the left colon (*P*=0.005, *F*=7.76, df=1871) were confirmed as having an independent association with FOBT Hb content.

## DISCUSSION

The present study is based on a relatively large consecutive series, observed within a single population-based screening programme, and followed up according to the same protocol. Immunological-FOBT was based on the same assay over the study period, and processed by a single laboratory: these conditions allow for a reliable estimate of the association of faecal Hb content to the presence of colonic lesions in I-FOBT+ subjects undergoing colonoscopy assessment.

Immunological-FOBT proved to be a rather specific test, with a 41.5% PPV for the presence of CRC or adenoma among assessed subjects, definitely a higher figure as compared to other cancer screening programmes, such as mammographic or cytological screening, where the PPV of a positive screening test does not currently exceed 20%. It is implied that such a high PPV is at least partially due to the exit from screening of bleeders with benign disease undergoing negative colonoscopy, but other reasons, such as a selection of high-risk subjects among screening compliers, cannot be excluded.

Increased faecal Hb content was significantly associated with increasingly severe colonic lesions, confirming previous reports on this subject (Edwards, 2005; Vilkin *et al*, 2005). A continuous trend of increasing Hb content was evident, from subjects with negative colonoscopy outcome, to subjects with benign colonic abnormalities other than adenoma, to subjects with adenoma as the most severe lesion, and finally to subjects with CRC. Among subjects with adenoma, faecal Hb content was higher for high-risk subcategories, such as larger size, presence of severe dyplasia or villous component. To our knowledge, this is the first study reporting the association of faecal Hb content to single adenoma characteristics, whereas previous studies grouped adenomas into low- and high-risk categories (Edwards, 2005; Vilkin *et al*, 2005). The higher average faecal Hb content recorded for adenomas in the left colon may be likely explained with a higher mechanical action of stool in this section.

Multivariate analysis confirmed an independent and significant association with higher faecal Hb content for patient's age, adenoma location in the left colon, and adenoma size, the latter variable showing the strongest association. This, combined with the association of Hb content with adenoma characteristics, and with the adoption of a 100 ng ml^−1^ Hb positivity threshold, allowed for a favourable advanced/not advanced adenoma ratio of approximately 3 : 1 (669/221), as compared to approximately 1 : 1 when guaiac FOBT was employed (Castiglione, 1996).

Immunological-FOBT positivity rate in the present experience was 3.73%. Positivity rate owing to non-neoplastic bleeding is likely to be reduced at repeat screening, as I-FOBT+ colonoscopy negative subjects, who may have a tendency for persistent spurious intestinal bleeding, exit the screening process (for 10 years in the Florence screening programme). Thus, biennial I-FOBT screening from 50 to 70 years of age would imply approximately 20–30% of screened subjects being I-FOBT+ and invited to undergo colonoscopy over a 20 years period. When such figures are compared to a 20% intention-to-screen-based mortality reduction reported by FOBT screening trials, the hypothesis of FOBT screening being effective only through colonoscopies recommended for spurious FOBT positivity is hardly acceptable.

Our findings show that I-FOBT is a specific screening test to detect CRC and adenoma. Immunological-FOBT is associated with a relatively low (3.73%) positivity rate, with a rather high (41.5%) positive predictive value for colonic neoplasms, and faecal Hb content is significantly related to the presence of those lesions (CRC and high-risk adenomas) screening is aimed at detecting.

## Figures and Tables

**Table 1:** Average FOBT Hb content observed in studied subjects according to patient characteristics and colonoscopy outcome

**Variable**	**Category**	**Average FOBT Hb content (ng m^−l^)**	**s.d.**	**Total cases**	***P*-value (*t* test/ANOVA)**
Gender	Male	466.0	494.6	1488	0.003
	Female	438.8	631.7	1109	
					
					
Age (years)	48–54	404.2	587.9	463	0.02
	55–59	473.3	546.8	525	
	60–64	497.3	644.9	681	
	65–69	438.2	476.0	730	
	70–76	433.4	445.9	198	
					
*Diagnostic assessment outcome (most severe lesion)*					
Cancer	—	817.0	645.2	191	0.000
Adenoma	Any	509.2	528.6	890	
Benign, other	—	407.5	610.7	985	
Negative	—	319.0	369.1	531	
					
Haemorroids	Present	439.6	695.8	742	0.002
	Absent	460.3	491.3	1855	
					
Diverticula	Present	475.6	628.4	598	0.688
	Absent	448.0	534.2	1999	

ANOVA=analysis of variance; FOBT=faecal occult blood testing; Hb=haemoglobin.

**Table 2:** Average FOBT Hb content observed in studied subjects with adenoma as the most severe lesion according to different subcategories (when multiple, only the adenoma largest in size or with most severe features was considered)

**Variable**	**Category**	**Average FOBT Hb content (ng m^−l^)**	**s.d.**	**Total cases**	***P*-value (*t* test/ANOVA)**
Size (cm)	<1	406.9	489.0	341	0.000
	1–2	486.0	476.5	279	
	>2	662.4	590.9	270	
					
Severe dysplasia	Present	615.0	544.1	175	0.0001
	Absent	483.3	521.9	715	
					
Histological type	Villous	559.8	544.2	487	0.0002
	Tubular	448.0	503.0	403	
					
Site (colon)	Left	520.5	505.9	744	0.0037
	Right	448.2	639.1	135	

ANOVA=analysis of variance; FOBT=faecal occult blood testing; Hb=haemoglobin.

**Table 3:** Results of multivariate analysis (ANOVA) of the association of different patient characteristics and colonoscopy outcome to average FOBT Hb content among subjects with adenoma

**Source**	**df**	**F**	***P*-value**
Model	12	6.77	0.000
Age (48–54, 55–59, 60–64, 65–69, 70–76 years)	4	2.34	0.05
Gender (male *vs* female)	1	0.35	0.55
Largest adenoma size (<1, 1–2, >2 cm)	2	19.84	0.000
Presence of adenoma with severe dysplasia	1	0.60	0.43
Presence of adenoma with villous component	1	0.30	0.58
Adenoma localisation (left *vs* right colon)	1	6.65	0.01
Presence of haemorroids	1	0.07	0.79
Presence of diverticula	1	0.59	0.44
